# California State Veterinary Association

**Published:** 1896-02

**Authors:** Thomas Maclay, H. A. Spencer

**Affiliations:** Secretary pro tem.; President pro tem.


### CALIFORNIA STATE VETERINARY ASSOCIATION.

THE annual meeting was held in the lecture rooms of the California Veterinary College, San Francisco, on December 11, 1895. The meeting was called to order at 2 P.M. by the Secretary, Dr. R. A. Archibald, who stated that as the President and Vice-President were both unavoidably absent, it would be necessary to elect a President *pro tem.* Whereupon Dr. H. A. Spencer, of San Jose, was unanimously chosen. The roll was called, and the following members answered to their names: Profs. Skaife and Egan, Drs. Archibald Spencer, Sr., Spencer, Jr., Pierce, Fabbi, Forrest, Maclay, Jackson, Hagerty, and Willard; visitors, Professors Steers and Cunningham, and the students of the California Veterinary College. Professor Skaife, Dean of the Faculty of the California Veterinary College, tendered the Association a hearty welcome

The minutes of the previous meeting were read and approved.

The Secretary stated that it would be in order for the Chair to appoint a committee as provided for in a resolution adopted June 12, 1895. By request said resolution was read. It was moved by Dr. Maclay and seconded by Dr. Egan that the motion whereby said resolution was adopted be rescinded; so ordered. The subject-matter of the resolution was taken up and considered, and several amendments proposed. It was moved by Dr. Maclay and seconded by Dr. Egan that a committee of one be appointed by the Chair to make the required amendments; so ordered; whereupon the Chair appointed Dr. Maclay as said committee, and he reported as follows: "Your special committee appointed to make required changes in resolution introduced on June 12, 1895, beg to report that all of said changes have been made, and the resolution herewith returned." On motion, the report of the commitee was received, and the Secretary requested to read the resolution as offered, *and as amended by the committee:*

"*Whereas*, The live-stock interests of this State are suffering from the malignant influences of contagious and infectious diseases; and, *whereas*, the health of the public of this great State is also jeopardized by the presence of said contagious and infectious diseases which affect the domestic animals; and, *whereas*, we, the members of the California State Veterinary Medical Association realize that this state of affairs is due to the fact that there are no laws on the statute books of this State sufficiently adequate to control the ravages of these contagious and infectious diseases, therefore, be it *Resolved*, That the presiding officer of this meeting be, and is hereby authorized and requested to appoint a committee of three members of this Association, whose duty it shall be to wait on or communicate with the Governor of this State, with a view of prevailing upon him to appoint a commission to consist of two veterinarians, one physician, one attorney, and one dairyman or stock-raiser, who shall receive no compensation, and whose duty it shall be to devise ways and means whereby the public health and the live-stock interests of this State may best be protected from the ravages of contagious and infectious animal diseases, such as anthrax, tuberculosis, glanders, hog cholera, etc."

It was moved by Dr. Maclay and seconded by Dr. Egan that the resolutions as amended be adopted; so ordered. Whereupon the President *pro tem.* appointed Drs. Mackay, Skaife, and Archibald a committee as provided for in the foregoing resolution.

Secretary Dr. Archibald read his annual report for the year ending December 1, 1895. On motion by Dr. Price, said report was received and ordered filed.

The Treasurer being absent, the hearing of his annual statement was postponed until the next quarterly meeting of the Association.

An application for membership from Professor Steers, of the California Veterinary College, was read and referred to the Board of Examiners, with the request that said application be acted upon before the end of the present convention.

The Chair stated that before proceeding with the election of officers it would be necessary to nominate some gentleman for the office of President; further, that nominations for all other officers were still open. Professor Skaife, of San Francisco, was elected President; Dr. H. Lemke, Bakerfield, Vice-President; Dr. D. F. Fox, Sacramento, Secretary; Dr. E. F. Pierce, Oakland, Treasurer; and Drs. Maclay, Egan, Spencer, Sr., McCollum, and Graham, Board of Examiners. The newly elected President, Prof. Skaife, thanked the members for the honor conferred, and asked President *pro tem.* Spencer to continue as chairman for the remainder of the session, which Dr. Spencer kindly consented to do. The Secretary-elect, Dr. Fox, not being present, and Dr. Archibald having no desire to continue the duties of Secretary, Dr. Maclay was requested and consented to act as Secretary for the remainder of the session.

The subject of delinquencies for dues and assessments was taken up and considered. Dr. Egan moved that the Secretary be instructed to notify all members of the amount of their arrearages, and inform them if said amount was not paid on or before the second Wednesday of March, 1896, that their names be referred to the Board of Directors, with instructions to inforce Section 2 of Article VII. of the By-laws: "Any member two years in arrears for dues or assessments shall have his name dropped from the roll of membership;" so ordered.

A very interesting paper was read by Dr. H. F. Spencer, of San Jose, on "Stomatitis Pustulosæ Contagiosæ." A lively discssion followed.

Dr. Maclay moved that the Association take a recess until 7.30 P.M.; so ordered. At 7.30 o'clock the Association reassembled, President *pro tem.* Spencer in the chair.

Profesor Skaife read a very excellent paper on "Physiology," which was handled in a masterly manner, and provoked a warm discussion.

Dr. R. A. Archibald read a very learned and interesting paper on "Contraction of the Foot." This paper was accompanied by a large number of crayon drawings of the horse's foot in health and disease; also drawings of the various kinds of shoes for the relief of contraction. The essayist demonstrated that he had given the subject a great amount of study. The reading was followed by a very interesting discussion, the majority of the members present taking part.

Dr. Maclay moved that the thanks of the Association be tendered to Ex-Secretary Dr. R. A. Archibald for the very able manner in which he conducted the duties of his office during the past four years; so ordered.

The Board of Examiners reported favorably on the application of Professor Steers, and, on motion of Dr. Archibald, he was elected to membership.

It was moved by Professor Skaife, and seconded by Dr. Archibald, that Professor Cunningham, of the California Veterinary College, be elected an honorary member; adopted. Professor Cunningham being present, thanked the members for the honor, and concluded his remarks by stating that he desired to make investigations on the disease of "Azoturia," and to that end requested the members to furnish him with small quantities of the urine passed by animals suffering from the said affection.

It was moved by Dr. Maclay, and seconded by Dr. Jackson, that the thanks of this Association be tendered to Professor Skaife, Dr. R. A. Archibald, and Dr. H. F. Spencer, for the very learned and interesting papers read at this session; further, that said gentlemen be requested to furnish the Secretary with copies of their papers, to be forwarded to the veterinary journals of America; so ordered.

It was moved by Dr. Maclay, and seconded by Dr. Forrest, that the election of Dr. Creely be rescinded, on the grounds that his election was against the best interests of the Association, and his non-compliance with the By-laws in regard to fees and dues and Code of Ethics; so ordered.

It was moved by Dr. Archibald, and seconded by Dr. Pierce, that the thanks of this Association be tendered the faculty of the California Veterinary College for the use of the lecture-hall and other privileges; so ordered. Professor Skaife responded, by stating that the faculty earnestly hoped the Association would hold their next meeting in the same place.

Mr. Welch, on behalf of himself and fellow-students of the California Veterinary College, thanked the Association for the privilege granted them during the session.

President Skaife announced that Drs. Pierce, Egan, and Jackson would read papers at the next meeting of the Association.

The Chair announced that on the following day (Thursday, December 12th) at 10 A.M., a clinical entertainment would be held at the College Hospital, conducted by Dr. R. A. Archibald.

Adjourned to meet on the second Wednesday in March, 1896, at the California Veterinary College, San Francisco.

THOMAS MACLAY, Secretary *pro tem.*

H. A. SPENCER, President *pro tem.*

